# Rheumatoid Arthritis and Cardio-Cerebrovascular Disease: A Mendelian Randomization Study

**DOI:** 10.3389/fgene.2021.745224

**Published:** 2021-10-21

**Authors:** Shizheng Qiu, Meijie Li, Shunshan Jin, Haoyu Lu, Yang Hu

**Affiliations:** ^1^School of Life Sciences and Technology, Harbin Institute of Technology, Harbin, China; ^2^Department of Neurology, Xuanwu Hospital, Capital Medical University, Beijing, China; ^3^General Hospital of Heilongjiang Province Land Reclamation Bureau, Harbin, China

**Keywords:** Mendelian randomization, genome-wide association studies, rheumatoid arthritis, cardiovascular disease, inverse-variance weighted

## Abstract

Significant genetic association exists between rheumatoid arthritis (RA) and cardiovascular disease. The associated mechanisms include common inflammatory mediators, changes in lipoprotein composition and function, immune responses, etc. However, the causality of RA and vascular/heart problems remains unknown. Herein, we performed Mendelian randomization (MR) analysis using a large-scale RA genome-wide association study (GWAS) dataset (462,933 cases and 457,732 controls) and six cardio-cerebrovascular disease GWAS datasets, including age angina (461,880 cases and 447,052 controls), hypertension (461,880 cases and 337,653 controls), age heart attack (10,693 cases and 451,187 controls), abnormalities of heartbeat (461,880 cases and 361,194 controls), stroke (7,055 cases and 454,825 controls), and coronary heart disease (361,194 cases and 351,037 controls) from United Kingdom biobank. We further carried out heterogeneity and sensitivity analyses. We confirmed the causality of RA with age angina (OR = 1.17, 95% CI: 1.04–1.33, *p* = 1.07E−02), hypertension (OR = 1.45, 95% CI: 1.20–1.75, *p* = 9.64E−05), age heart attack (OR = 1.15, 95% CI: 1.05–1.26, *p* = 3.56E−03), abnormalities of heartbeat (OR = 1.07, 95% CI: 1.01–1.12, *p* = 1.49E−02), stroke (OR = 1.06, 95% CI: 1.01–1.12, *p* = 2.79E−02), and coronary heart disease (OR = 1.19, 95% CI: 1.01–1.39, *p* = 3.33E−02), contributing to the understanding of the overlapping genetic mechanisms and therapeutic approaches between RA and cardiovascular disease.

## Introduction

Cardiovascular disease remains the leading cause of human death, with an estimated 17.3 million people worldwide dying of cardiovascular disease each year, which is expected to increase to 23.6 million by 2030 (Laslett et al., 2012; Smith et al., 2012; Leong et al., 2017). Epidemiological studies have shown that the occurrence of cardiovascular disease is caused by various factors, with obesity, diabetes, smoking, hyperlipidemia, atherosclerosis, hypertension, and blood viscosity being its potential risk factor (Leong et al., 2017; Xu et al., 2019). Importantly, traditional cardiovascular disease risk factors account for a large proportion of rheumatoid arthritis (RA) (An et al., 2016). RA patients were 48% more likely to have cardiovascular disease than normal people and a 50% higher incidence of cardiovascular disease-related mortality (Avina-Zubieta et al., 2008, 2012; Sokka et al., 2008; England et al., 2018). However, most of the previous studies have examined the association between RA and atherosclerosis and congestive heart failure, ignoring other phenotypes of heart disease and vascular problems (England et al., 2018). Moreover, the exact causality is still unknown.

Mendelian randomization (MR) could estimate causality without bias, which has been used in previous studies to explore the association between phenotypes (Jansen et al., 2014; Smith et al., 2017; Hemani et al., 2018; Cheng et al., 2019b, 2021; Zhuang et al., 2019; Qiu et al., 2021). Causality between multiple metabolic characteristics, nutrient elements, and common diseases with cardiovascular disease have been demonstrated (Ference et al., 2017; Larsson et al., 2017, 2020; Yeung et al., 2018; Rosoff et al., 2020; Arvanitis et al., 2021). However, strong evidence linking RA to cardiovascular disease is still lacking. Herein, we mainly selected inverse-variance weighted (IVW), weighted median, and MR-Egger methods for MR analysis. We provided strong evidence that RA contributed to six vascular-/heart problem-related phenotypes, which could be of great significance for clinical disease prevention and treatment.

## Materials and Methods

### Genome-Wide Association Study Dataset Sources

We obtained large-scale genome-wide association study (GWAS) summary datasets from the United Kingdom Biobank on RA and six cardiovascular disease phenotypes, and all of the participants were of European ancestry. From 2006 to 2010, the United Kingdom Biobank Assessment Center recruited 386,005 participants from the United Kingdom to participate in self-reporting of non-cancer illness (Li et al., 2015; Sun et al., 2019). RA GWAS (462,933 cases and 457,732 controls) was derived from the non-cancer illness study. At the same time, the United Kingdom Biobank Assessment Center carried out the study of vascular/heart problems diagnosed by doctors, covering 501,555 participants. These heart or vascular problems included age angina (461,880 cases and 447,052 controls), age high blood pressure (461,880 cases and 337,653 controls), age stroke (7,055 cases and 454,825 controls), and age heart attack (10,693 cases and 451,187 controls). In addition, we supplemented two other United Kingdom Biobank studies on coronary heart disease (CHD) (361,194 cases and 351,037 controls) and abnormalities of heartbeat (461,880 cases and 361,194 controls).

### Quality Control and Identifying Genetic Instruments

In order to enhance the statistical power of genetic variants, we deleted single-nucleotide polymorphisms (SNPs) with a minor allele frequency (MAF) < 1%. Moreover, we removed variants with physical distance less than 10,000 kb and *R*^2^ < 0.001 to avoid linkage disequilibrium (LD). For preprocessed exposure (RA) data, we selected genetic variants that passed genome-wide association threshold (*p* < 5E−08) as instrumental variables (IVs) to satisfy IV assumption 1 of MR analysis: variants should be strongly associated with exposure (RA) (Hemani et al., 2018).

### Two-Sample Mendelian Randomization Analysis

In the absence of individual-level data, we used MR, a powerful statistical method, to infer the causality between two phenotypes. MR analysis eliminates the need to consider confounders and reverse causality. Two-sample MR requires that the samples of exposure and outcome be independent, which greatly expands the application range of MR (Hemani et al., 2018). Details of MR analysis have been described in previous reports (Davey Smith and Hemani, 2014; Bowden et al., 2015, 2016; Yavorska and Burgess, 2017; Cheng et al., 2018, 2019b; Hemani et al., 2018; Hu et al., 2020, 2021; Qiu et al., 2021). Herein, we first aligned alleles on the forward strand and harmonized SNP effects of exposure and outcome (Ong et al., 2021). If the variant in IVs was lacking in outcome, we allowed the proxy SNP with a strong LD to replace it (Hemani et al., 2018). Subsequently, we performed the inverse-variance weighted (IVW) estimator to estimate the association between RA and vascular/heart disease (Burgess and Thompson, 2017). In the case of certain invalid instruments or directional pleiotropy bias, the weighted median and MR-Egger estimators could help to make further judgment (Bowden et al., 2017; Burgess and Thompson, 2017; Hartwig et al., 2017; Cheng, 2019; Cheng et al., 2019a). Finally, we carried out reverse MR analysis to evaluate the evidence for reverse causal association.

### Sensitivity Analysis

We performed a series of sensitivity tests to ensure that our results were robust, including heterogeneity tests to assess heterogeneity between IVs, leave-one-out analysis (Wei et al., 2019, 2021; Govindaraj et al., 2020; Hasan et al., 2021) to assess whether a single SNP over-drove outcome, and funnel plots and MR-Egger to assess potential horizontal pleiotropy (Bowden et al., 2017; Hartwig et al., 2017; Rosoff et al., 2020). The statistical tests for MR analysis were undertaken using the R package of meta and TwoSampleMR (Hemani et al., 2018). The statistically significant association is defined as *p* < 0.05.

## Results

### Association of Rheumatoid Arthritis With Cardiovascular Disease

**FIGURE 1:**
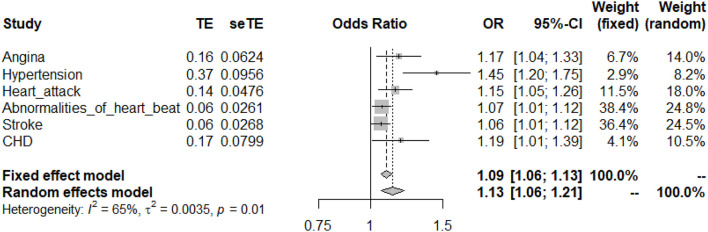
Mendelian randomization (MR) analysis between rheumatoid arthritis (RA) and six cardiovascular diseases. TE, treatment effects (β); se TE, standard error of treatment effect (se).

Eight genetic variants were used as IVs to evaluate the association between RA and cardiovascular disease (Table 1). Two SNPs needed to be proxied, but we lacked evidence for the presence of wrong effect alleles, strand issues, palindromic SNPs, and incompatible alleles. Due to the heterogeneity in some studies, we preferred to use the random-effects model (Table 2). By performing IVW analysis, we confirmed the causality of RA with age angina (OR = 1.17, 95% CI: 1.04–1.33, *p* = 1.07E−02), hypertension (OR = 1.45, 95% CI: 1.20–1.75, *p* = 9.64E–05), age heart attack (OR = 1.15, 95% CI: 1.05–1.26, *p* = 3.56E–03), abnormalities of heartbeat (OR = 1.07, 95% CI: 1.01–1.12, *p* = 1.49E−02), stroke (OR = 1.06, 95% CI: 1.01–1.12, *p* = 2.79E−02), and CHD (OR = 1.19, 95% CI: 1.01–1.39, *p* = 3.33E−02) (Figure 1). Detailed MR results are shown in the Supplementary Material. No evidence of reverse causality existed in any of the studies. Thus, RA made a significant contribution to common phenotypes associated with vascular or cardiac problems.

**TABLE 1:** Characteristics of eight genetic variants as instrumental variables (IVs).

SNP	Chr	Pos	Effect allele	Other allele	Beta	EAF	SE	*p*
rs185320691	6	32,490,292	C	G	0.0076	0.10	0.00040	2.30E−82
rs28559870	6	31,377,974	T	C	0.0017	0.18	0.00029	4.10E−09
rs35175534	6	32,531,108	C	A	0.0076	0.14	0.00035	3.50E−105
rs460568	6	33,232,025	T	C	0.0019	0.17	0.00029	4.10E−11
rs6679677	1	114,303,808	A	C	0.0031	0.10	0.00036	4.90E−18
rs7731626	5	55,444,683	A	G	−0.0015	0.38	0.00023	2.00E−11
rs7760841	6	32,574,868	T	C	0.0083	0.17	0.00030	7.29E−172
rs9265076	6	31,287,765	T	C	0.0021	0.38	0.00026	1.10E−15

*Beta is the estimated effect size for the effect allele; Beta > 0 and Beta < 0 means that this effect allele could increase and reduce RA risk, respectively. EAF, effect allele frequency.*

**TABLE 2:** The results of Mendelian randomization (MR) sensitivity analysis.

Phenotypes Methods	Angina	Hypertension	Heart attack	Abnormalities of heartbeat	Stroke	Coronary heart disease
MR-Egger	0.494	0.090	0.373	0.481	0.448	0.306
Heterogeneity tests	0.010	0.472	0.038	0.293	0.566	0.003
Leave-one-out analysis	0.0107	9.64E−05	0.00356	0.0149	0.0279	0.0333

*The values in the table are all *p*-values.*

*MR-Egger: *p* > 0.05 means that the original hypothesis is rejected, and horizontal pleiotropy (non-zero intercept) has no significant effect on the results.*

*Heterogeneity tests: *p* > 0.05 means no heterogeneity.*

*Leave-one-out analysis: *p* < 0.05 means that no single single-nucleotide polymorphism (SNP) over-drives the overall results.*

### Sensitivity Analysis

**FIGURE 2:**
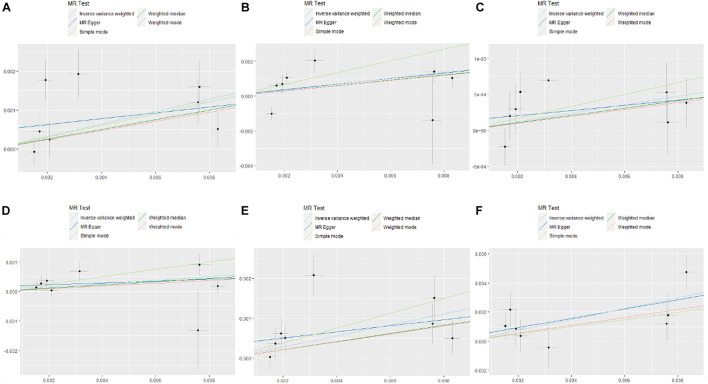
MR tests of RA with angina, hypertension, heart attack, abnormalities of heartbeat, stroke, and coronary heart disease. The estimate of intercept can be interpreted as an estimate of the average pleiotropy of all single-nucleotide polymorphisms (SNPs), and the slope coefficient provides an estimate of the bias of the causal effect. **(A)** Angina. **(B)** Hypertension. **(C)** Heart attack. **(D)** Abnormalities of heartbeat. **(E)** Stroke. **(F)** Coronary heart disease.

**FIGURE 3:**
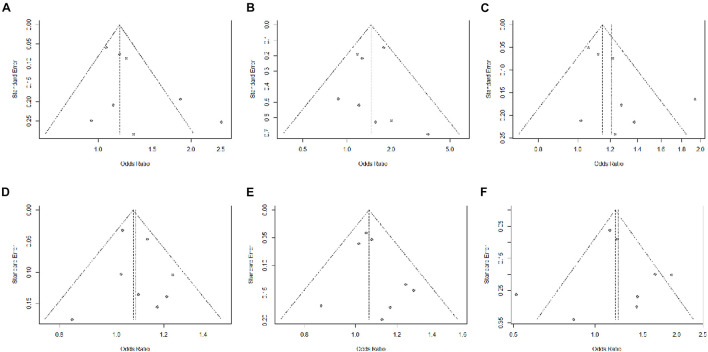
Funnel plots of RA with angina, hypertension, heart attack, abnormalities of heartbeat, stroke, and coronary heart disease. The x-axis represents odds ratio (OR), and the y-axis represents standard error (se). **(A)** Angina. **(B)** Hypertension. **(C)** Heart attack. **(D)** Abnormalities of heartbeat. **(E)** Stroke. **(F)** Coronary heart disease.

Unlike IVW, MR-Egger allows horizontal pleiotropy between IVs and exposure and outcome, and the weighted median allows a more powerful variant to have a greater impact on the overall result (Bowden et al., 2017; Hartwig et al., 2017; Qiu et al., 2021). Other MR calculation methods are a powerful supplement to IVW, especially when the IV assumptions of MR framework is not satisfied perfectly. In all methods, our results were robust, with small intercepts and high *p*-values in MR-Egger, which meant that horizontal pleiotropy made almost no effect on the results (Figure 2 and Table 2). According to funnel plots, rs7731626 and rs460568 in angina, and rs6679677 and rs9265076 in CHD, there existed a certain horizontal pleiotropy; however, little influence affected the overall results (Figure 3). Moreover, no single SNP over-drove the overall results.

## Discussion

In this study, we carried out MR analysis to demonstrate that RA was positively associated with six heart and vascular diseases. Observational studies evaluated that the patients with RA had a significantly increased risk of cardiovascular disease (An et al., 2016; Crowson et al., 2018). Crowson et al. (2018) followed up 5,638 RA patients for 5.8 years and found that about 30% of them eventually developed cardiovascular disease. RA might increase the risk of the six heart and vascular diseases we mentioned at the same time (Kitas and Gabriel, 2011; Dougados et al., 2014; Hadwen et al., 2021). Thus, we subdivided the phenotypes of cardiac and vascular diseases to provide different risk values, which might be a significant help for the clinical cotreatment of RA and cardiovascular disease.

Our study may have many advantages over previous observational studies. First, we used seven large-scale GWAS datasets. RA GWAS alone involved more than 900,000 participants. The seven studies were all from European descent, avoiding potential population stratification. Second, MR greatly avoids the influence of confounding factors and reverse causality because the alleles of the SNP site are randomly assigned much earlier than the occurrence of any potential confounding factors. Third, we applied a variety of MR methods to jointly verify the robustness of the results. When some instruments were unavailable or horizontal pleiotropy, an unbiased causal estimate could still be given.

However, certain limitations existed in our study. First, there may be overlap of some samples in RA GWAS and cardiovascular disease GWAS, which leads to weak instruments bias (Pierce and Burgess, 2013). In addition, some of the genetic variants have a certain degree of heterogeneity or horizontal pleiotropy, such as rs7731626 and rs9265076, in the analysis of RA and angina. For population stratification from gender, age, and ancestry, inappropriate proxy SNP may be the potential reasons for them.

In conclusion, we explored the causality between RA and six cardio-cerebrovascular diseases for the first time, and all the results showed the risk effect of RA. Eight variants as IVs may be the link between RA and cardiovascular disease. We expect to find out the genetic association between chronic diseases more deeply in the future.

## Data Availability Statement

The original contributions presented in the study are included in the article/Supplementary Material, further inquiries can be directed to the corresponding author/s.

## Author Contributions

SQ and LH designed the study, analyzed the data, and wrote the manuscript. ML and SJ revised the manuscript. YH supervised the research and revised the manuscript. All authors contributed to the article and approved the submitted version.

## Conflict of Interest

The authors declare that the research was conducted in the absence of any commercial or financial relationships that could be construed as a potential conflict of interest.

## Publisher’s Note

All claims expressed in this article are solely those of the authors and do not necessarily represent those of their affiliated organizations, or those of the publisher, the editors and the reviewers. Any product that may be evaluated in this article, or claim that may be made by its manufacturer, is not guaranteed or endorsed by the publisher.
