# Supplementary material for: Rheumatoid Arthritis and Cardio-Cerebrovascular Disease: A Mendelian Randomization Study
Source: Front Genet. 2021 Oct 21;12:745224. doi: 10.3389/fgene.2021.745224 (PMC8567962; doi:10.3389/fgene.2021.745224)
Supplement: Supplementary file 1 [file Data_Sheet_1.docx]

**Supplementary material**

**Supplementary Figure 1. Mendelian randomization analysis of RA and angina.**


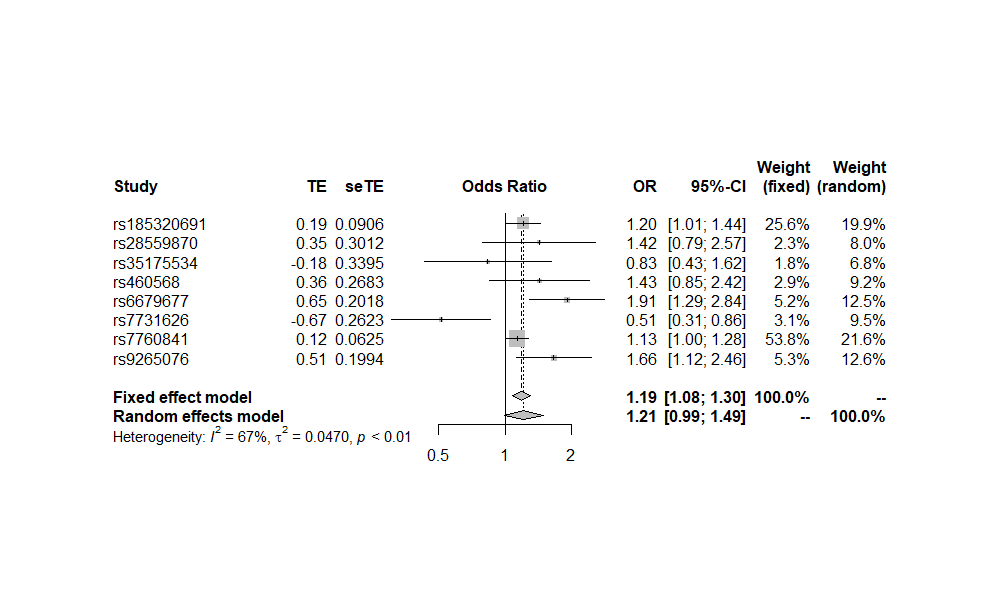


TE: treatment effects (ꞵ); se TE: Standard error of treatment effect (se).

**Supplementary Figure 2. Mendelian randomization analysis of RA and hypertension.**


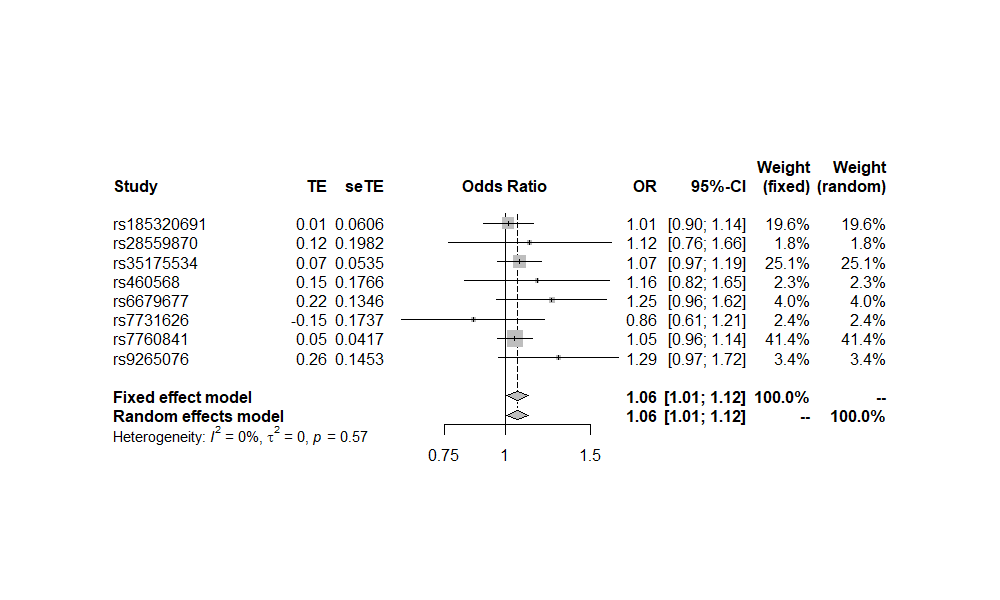


TE: treatment effects (ꞵ); se TE: Standard error of treatment effect (se).

**Supplementary Figure 3. Mendelian randomization analysis of RA and heart attack.**


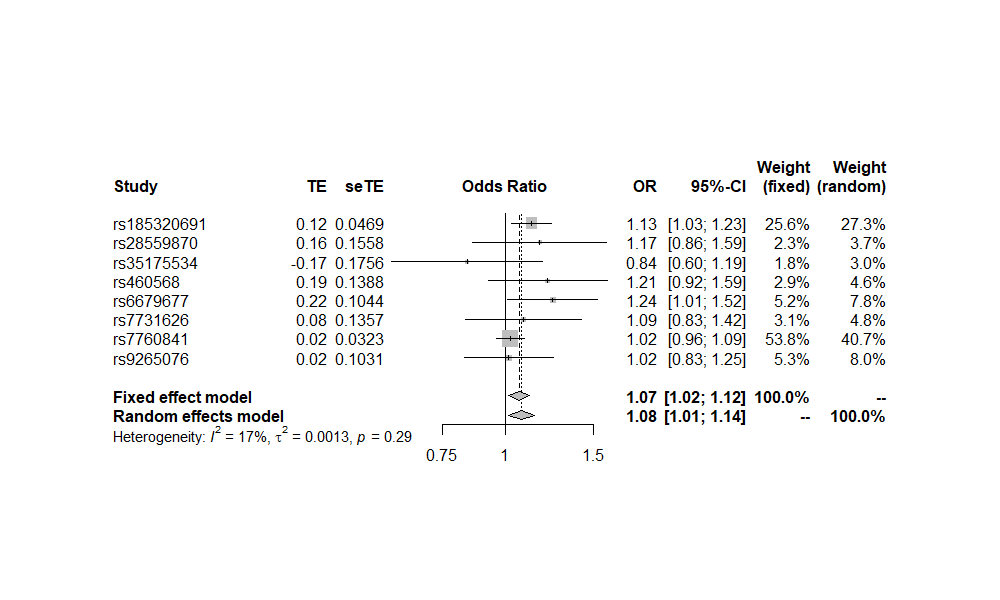
 TE: treatment effects (ꞵ); se TE: Standard error of treatment effect (se).

**Supplementary Figure 4. Mendelian randomization analysis of RA and abnormalities of heart beat.**


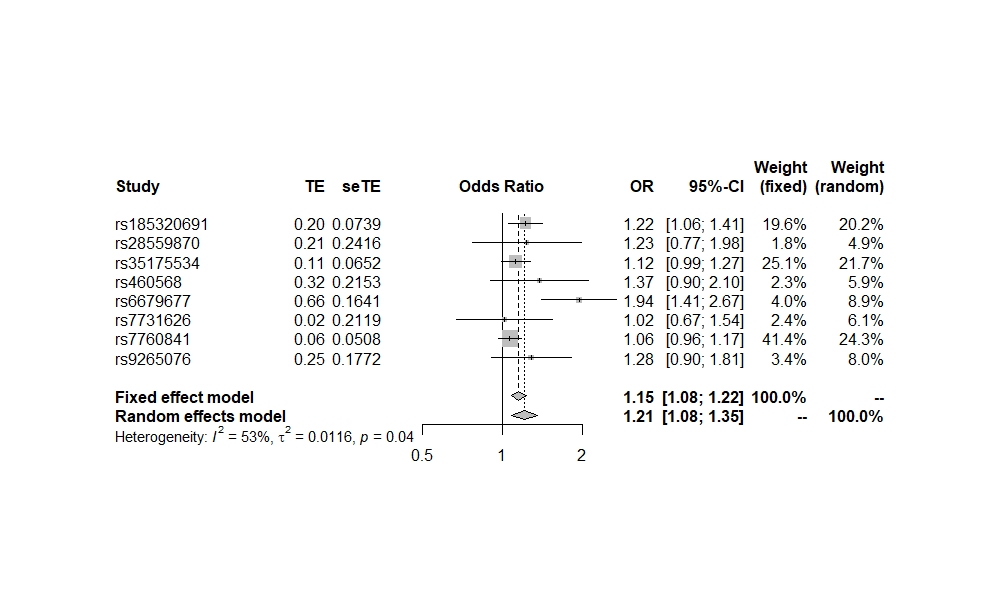


TE: treatment effects (ꞵ); se TE: Standard error of treatment effect (se).

**Supplementary Figure 5. Mendelian randomization analysis of RA and stroke.**


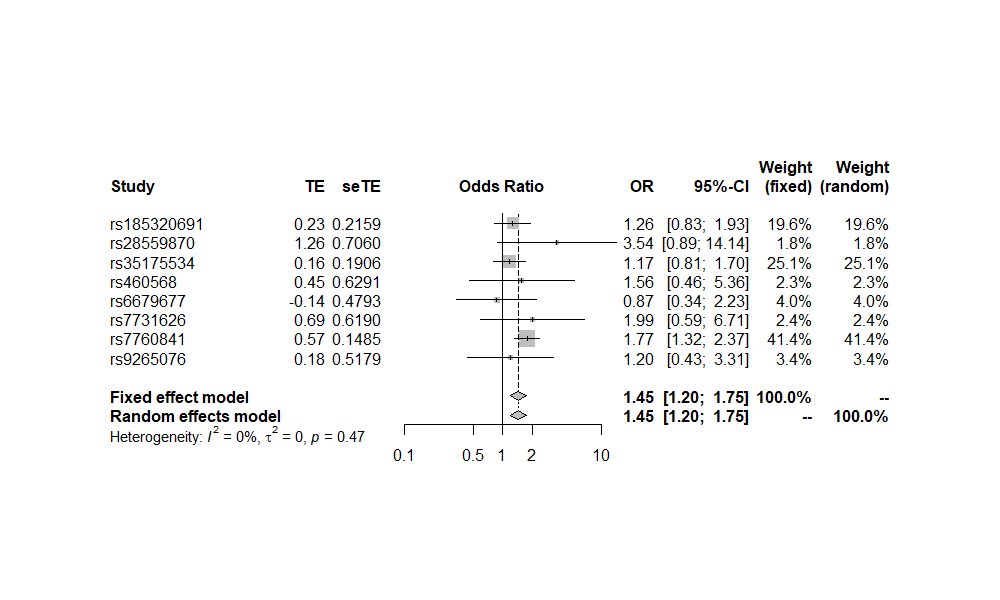
 TE: treatment effects (ꞵ); se TE: Standard error of treatment effect (se).

**Supplementary Figure 6. Mendelian randomization analysis of RA and coronary heart disease.**


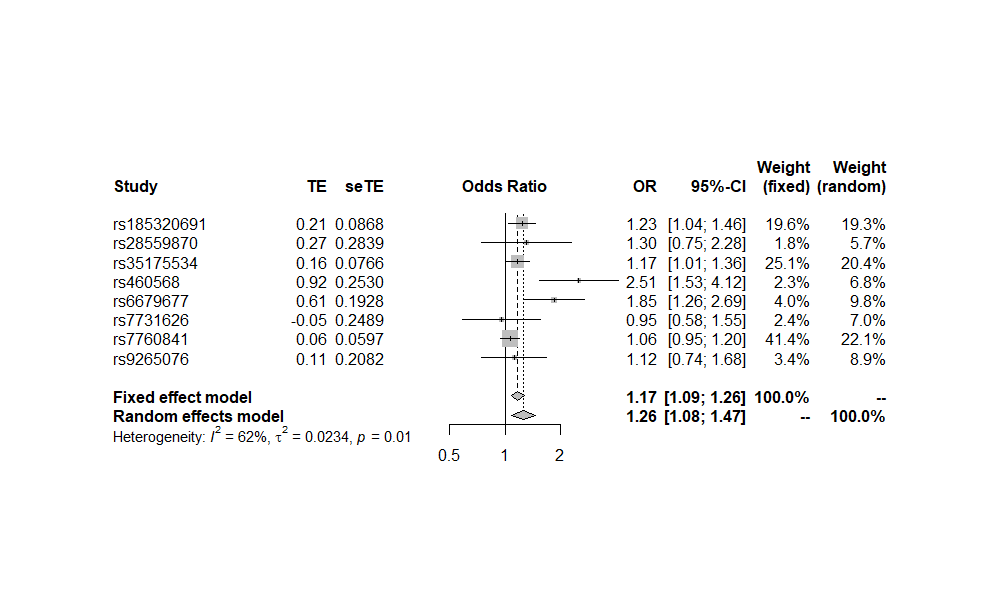
 TE: treatment effects (ꞵ); se TE: Standard error of treatment effect (se).
